# Comparison of hydrophobicity scales for predicting biophysical properties of antibodies

**DOI:** 10.3389/fmolb.2022.960194

**Published:** 2022-08-31

**Authors:** Franz Waibl, Monica L. Fernández-Quintero, Florian S. Wedl, Hubert Kettenberger, Guy Georges, Klaus R. Liedl

**Affiliations:** ^1^ Department of General, Inorganic and Theoretical Chemistry, University of Innsbruck, Innsbruck, Austria; ^2^ Large Molecule Research, Roche Pharma Research and Early Development, Roche Innovation Center Munich, Penzberg, Germany

**Keywords:** hyrophobicity, hydrophobicity scale, hydrophobic interaction chromatography (HIC), antibodies, developability, developability prediction, antibody structure

## Abstract

While antibody-based therapeutics have grown to be one of the major classes of novel medicines, some antibody development candidates face significant challenges regarding expression levels, solubility, as well as stability and aggregation, under physiological and storage conditions. A major determinant of those properties is surface hydrophobicity, which promotes unspecific interactions and has repeatedly proven problematic in the development of novel antibody-based drugs. Multiple computational methods have been devised for in-silico prediction of antibody hydrophobicity, often using hydrophobicity scales to assign values to each amino acid. Those approaches are usually validated by their ability to rank potential therapeutic antibodies in terms of their experimental hydrophobicity. However, there is significant diversity both in the hydrophobicity scales and in the experimental methods, and consequently in the performance of in-silico methods to predict experimental results. In this work, we investigate hydrophobicity of monoclonal antibodies using hydrophobicity scales. We implement several scoring schemes based on the solvent-accessibility and the assigned hydrophobicity values, and compare the different scores and scales based on their ability to predict retention times from hydrophobic interaction chromatography. We provide an overview of the strengths and weaknesses of several commonly employed hydrophobicity scales, thereby improving the understanding of hydrophobicity in antibody development. Furthermore, we test several datasets, both publicly available and proprietary, and find that the diversity of the dataset affects the performance of hydrophobicity scores. We expect that this work will provide valuable guidelines for the optimization of biophysical properties in future drug discovery campaigns.

## 1 Introduction

In recent years, antibodies and related formats have emerged as a major class of novel therapeutic proteins (Strohl and Strohl, 2012; Kaplon et al., 2020; Kaplon and Reichert, 2021; Mullard, 2021; Kaplon et al., 2022) with more than 120 approved therapeutic antibodies up to now (Raybould et al., 2020). Antibodies are characterized by their unique binding properties and their broad applicability. In particular, therapeutic antibodies have revolutionized the treatment of various diseases, such as cancer (Scott et al., 2012) and autoimmune diseases (Chan and Carter, 2010). Therapeutic antibodies are usually also the fastest answer to new medical challenges and viral threats, which has become apparent in the current SARS-CoV-2 pandemic (Chvatal-Medina et al., 2021).

In the development of therapeutic antibodies, it is important to avoid problems regarding the stability, aggregation, solubility, and immunogenicity. One driving force of those problems is the tendency of hydrophobic, i.e., apolar, regions on the surface to form interactions with each other. This phenomenon is called the hydrophobic effect and is driven by the release and entropy increase of water from the hydrophobic surface into bulk solution (Southall et al., 2002).

A plethora of methods have been used to investigate hydrophobicity of antibodies in-silico. A summary of those methods will be given in the subsection titled “In-silico methods.” However, it is unclear which methods and which hydrophobicity scales perform well at predicting a given experimental metric of hydrophobicity. Here, we compare between different methods and different scales to predict retention times from hydrophobic interaction chromatography (HIC). We hope that our findings will be useful to guide future efforts at in-silico optimization of potential biopharmaceuticals.

### 1.1 Hydrophobicity

Hydrophobicity is one of the most important predictors of developability when designing antibody-based drugs (Lauer et al., 2012; Hebditch et al., 2019). Large scientific efforts have been directed towards reducing hydrophobicity without affecting the binding capability (Jain et al., 2017a; Jain et al., 2017b; Raybould et al., 2019; Jo et al., 2020). It has been shown that hydrophobicity as well as surface charges contribute to self-aggregation in IgG-type antibodies (Esfandiary et al., 2015; Das et al., 2022). Hydrophobicity also leads to faster clearance in antibody-drug conjugates (ADCs) (Lyon et al., 2015). While some antibodies exhibit high hydrophobicity in their folded state, partial unfolding can lead to exposure of additional hydrophobic sidechains and accelerate aggregation (Amin et al., 2014).

Hydrophobicity of monoclonal antibodies (mAbs) is routinely quantified using Hydrophobic Interaction Chromatography (HIC) (Haverick et al., 2014; Wang et al., 2016), while their solubility may be assessed using, e.g., PEG precipitation assays (Gibson et al., 2011; Sormanni et al., 2017). Aggregation of mAbs is commonly assessed using size exclusion chromatography (Brusotti et al., 2018) or dynamic light scattering experiments combined with incubation under stress conditions like elevated temperature (Amin et al., 2014).

Within this work, we test different hydrophobicity scores for their ability to predict HIC retention times. We use both publicly available datasets (Jain et al., 2017b) and Roche-internal data. The novel HIC retention times are shown in the Supplementary Material. For antibodies from public sources, the sequence is shown together with the retention times.

### 1.2 Hydrophobicity of antibodies

Hydrophobic interactions of biomolecules are typically mediated by one or few hydrophobic surface patches. Experimentally, the interaction of proteins with a column in Hydrophobic Interaction Chromatography (HIC) is different for proteins with homogeneous or inhomogeneous hydrophobicity profiles (Mahn et al., 2009).

Here, we test hydrophobicity scores based on the whole surface as well as scores based only on the hydrophobic surface regions. Physically, the first option implies a process where the whole surface is desolvated and directly contacts the HIC column, while the latter implies that only the hydrophobic regions are desolvated. From literature, it is expected that the second process more closely describes the process of HIC.

Research on statistical mechanics of the hydrophobic effect suggests that hydrophobic patches or moieties need to exceed a certain minimum size to exhibit the full effect on the water properties (Acharya et al., 2010; Huang and Chandler, 2000; Acharya et al., 2010). Furthermore, the behavior of hydrophobic surfaces also depends on the experimental conditions. Additionally, hydrophobic interactions between biomolecules depend on many other effects such as shape complementarity between the interaction partners (Mahn et al., 2005), or entropic penalties due to reduced conformational flexibility in the bound state.

Aggregation propensity of biomolecules is often discussed in terms of the related concept of Aggregation Prone Regions (APRs). An APR is a part of a protein structure or sequence that has a high tendency to form aggregates when exposed to the protein surface (Wang et al., 2009). This concept has been linked to highly hydrophobic surface regions (Chennamsetty et al., 2010), but also to the propensity to unfold or form amyloid β-sheets (Willbold et al., 2021).

The treatment of hydrophobic patches in in-silico methods will be discussed below.

### 1.3 In-silico methods

A multitude of methods have been devised to predict the hydrophobicity of antibodies in-silico. Generally, they may be divided into structure-based and sequence-based approaches.

Sequence-based methods, such as CamSol (Sormanni et al., 2015) and many others (Conchillo-Sole et al., 2007; Tartaglia and Vendruscolo, 2008; Walsh et al., 2014), offer the highest computational speed, such that large numbers of sequences can be scanned. Furthermore, they may be used even in cases where the three-dimensional structure of a protein is unknown.

On the other hand, methods that incorporate the three-dimensional protein structure may yield more accurate results. Examples include AggScore (Sankar et al., 2018), the Spatial Aggregation Propensity (SAP) method (Chennamsetty et al., 2009), as well as AggreScan3D (Zambrano et al., 2015). When the structure is not known experimentally, these methods can still be applied in combination with in-silico structure prediction. However, we have shown previously (Waibl et al., 2021) that homology models are often insufficiently accurate to describe the surface hydrophobicity, at least when using descriptors based on molecular dynamics simulation of the surrounding water.

Many in-silico methods favor large hydrophobic surfaces by searching for continuous hydrophobic patches (Lijnzaad et al., 1996; Chemical Computing Group ULC, 2020). Alternatively, the hydrophobicity of nearby atoms can be incorporated using so-called hydrophobic potentials (Heiden et al., 1993). In this approach, hydrophobicity is mapped to the protein surface *via* a distance weighting function. Hydrophobicity scores are then computed either by summing the surface values or by searching for patches above a certain cutoff. This approach favors large hydrophobic patches, because the effect of a single hydrophobic atom can be negated by a more hydrophilic surrounding, while the values in a patch of several hydrophobic atoms add up favorably.

Another approach is to assign additional hydrophobicity to each atom or residue based on the hydrophobicity of nearby atoms (Manavalan and Ponnuswamy, 1978; Gromiha et al., 2013). Newer approaches, such as the Spatial Aggregation Propensity (SAP) (Chennamsetty et al., 2009; Lauer et al., 2012) or AggreScan3D (Zambrano et al., 2015) combine this approach with terms for the solvent-accessible surface area (SASA) to avoid contributions from the hydrophobic core of the protein. In the case of SAP, this is done by computing the sum of hydrophobicity values of surface-exposed side-chain atoms within a pre-defined cutoff radius (Chennamsetty et al., 2009; Chennamsetty et al., 2010).

While a lot of research has been conducted to elucidate the hydrophobic effect using all-atom explicit solvent simulations (Acharya et al., 2010; Waibl et al., 2021), those methods have not been widely adopted in biopharmaceutical research due to high computational demand.

### 1.4 Hydrophobicity scales

Both sequence-based and structure-based methods often treat hydrophobicity as an innate property of the atoms or amino acids that constitute the protein. The individual values are tabulated in hydrophobicity scales and can be based on experimental measurements or more detailed calculations.

A plethora of different hydrophobicity scales has been devised. Lienqueo et al. (2002) investigated the ability of different hydrophobicity scales to predict HIC retention times over a wide range of different proteins. They classify hydrophobicity scales into three groups. Firstly, direct scales are based on the transfer free energy of each amino acid between phases of different polarity, retention time in RP-HPLC, or other properties such as polarity or geometry. Secondly, indirect scales are based on the solvent-accessible surface area (SASA) or other spatial distributions of amino acids in a protein. Lastly, mixed scales are derived from several of those properties or incorporate previous hydrophobicity scales.

A review of experimental hydrophobicity scales has been presented by Biswas et al. (2003). The usage of hydrophobicity scales to predict HIC retention is summarized by Mahn et al. (2009), and applications on interactions between proteins and lipid membranes have been reviewed by MacCallum and Tieleman (2011). Another review focused on secondary structure prediction has been presented by Simm et al. (2016).

While most hydrophobicity scales provide hydrophobicity parameters on a per-residue basis, some scales have also been parameterized per-atom. Notable examples include the Wildman and Crippen (1999) parameters, which are designed to predict octanol-water partitioning coefficients (logP) and molecular refractivities of small molecules, as well as the scale by Eisenberg and Mclachlan (1986), which is fitted to the transfer free energy of amino acids between the outer and inner regions of a protein. While those scales clearly offer a higher structural resolution, they might also introduce additional inaccuracies, since properties of amino acids are not exactly equal to the sum of their atomic contributions.

In this study we aim to compare various hydrophobicity scales and descriptors regarding their ability to predict antibody surface hydrophobicity.

Since there are too many hydrophobicity scales in literature to test them all, we selected a representative set for the purpose of this work. The scales were selected based on three properties: they are widely used (Eisenberg, Kyte-Doolittle, Crippen, Wimley-White), they are specifically aimed at predicting HIC or RP-HPLC (Jain, Meek, Miyazawa), or they have been previously used for hydrophobicity of antibodies (Black-Mould). Additionally, we added the scale by Rose as an example of a scale based purely on the change in SASA on folding. In Table 1, we present the selected scales and classify them in terms of experimental data and their resolution (atomic or residue-based). In the Results section, we will compare these scales in terms of their ability to predict biophysical properties of monoclonal antibodies.

**TABLE 1:** Comparison of hydrophobicity scales that will be used in the present work.

Scale	Resolution	Principle	Notes	References
Bandyopadhyay-Methler	Residue	Local environment, Rekker coefficients (Rekker and Kort, 1979)		Bandyopadhyay and Mehler (2008)
Black-Mould	Residue	Rekker coefficients (Rekker and Kort, 1979)		Black and Mould (1991)
Eisenberg	Residue	Consensus of 5 previous scales		Eisenberg et al. (1982)
Jain	Residue	HIC retention		Jain et al. (2017a)
Kyte-Doolittle	Residue	Consensus of ΔG (water—vapor) and surface accessibility		Kyte and Doolittle (1982)
Meek	Residue	RP-HPLC retention	At pH 7.4	Meek (1980)
Miyazawa	Residue	Surface accessibility		Miyazawa and Jernigan (1985)
Rose	Residue	Fraction of surface area buried while folding		Eisenberg et al. (1982), Rose et al. (1985)
Wimley-White	Residue	ΔG (water—lipid bilayer)	Interface Scale	Wimley and White (1996)
Crippen	Atomic	LogP of small molecules		Wildman and Crippen (1999)
Eisenberg-dG	Atomic	Surface accessibility		Eisenberg and Mclachlan (1986)

### 1.5 Aim of this work

In the early stages of development, a fast in-silico method to estimate the hydrophobicity of antibodies is often desired. In this work, we test the performance of several existing methods combined with multiple hydrophobicity scales by predicting relative hydrophobicity of antibodies and comparing to experimental data from HIC. We compare the predictivity of those methods between multiple datasets with different sequence variability to test whether our scores work better for closely related sequences or for a broader selection of antibodies. We find that choosing an appropriate hydrophobicity scale is crucial to obtain good agreement with experiments. Additionally, the choice of the scoring function, the origin of the homology models, as well as conformational sampling, can influence the results. We also demonstrate that better correlation with experimental results can be expected using datasets with high sequence similarity, also in cases where no crystal structures are available.

## 2 Results

### 2.1 Comparison between the datasets

In this work, we investigate several sets of antibodies, which are described in detail in the Methods section. In short, the dataset by Jain et al. (2017b) (called the “Jain” dataset below) dataset contains 127 variable domains of antibodies which were approved or undergoing clinical trials at the time of publication. These variable domains were grafted on a constant IgG1 Fc domain for consistency. We further split this into a group where crystal structures are available (Jain-PDB) and one where we rely on homology models generated using DeepAb (Ruffolo et al., 2021) (Jain-models). The Roche-34 dataset contains a diverse set of Roche-internal in addition to publicly available antibodies. It overlaps with the Jain-Models in 3 cases. The Roche-127 contains a group of 127 closely related Roche-internal antibodies, for which no crystal structures are available.

To investigate how much of the antibody sequence space is spanned by our input datasets, we produced a tSNE (van der Maaten and Hinton, 2008) embedding of all investigated sequences combined with 2,000 randomly chosen sequences from the Observed Antibody Space database (Kovaltsuk et al., 2018; Olsen et al., 2022). The computational details are described in the Methods. The tSNE embedding produces a two-dimensional coordinate representation based on a distance matrix and aims to reproduce primarily the small distances. This means that groups of points in the embedding represent highly similar antibodies, while the distance between such groups is not necessarily representative of their similarity.

**FIGURE 1:**
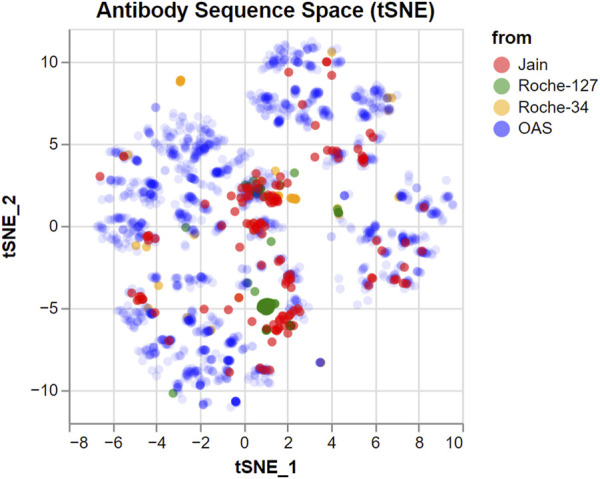
tSNE embedding of the sequence space covered by the datasets used in this study. 2,000 random sequences from the OAS dataset are added for comparison, with a lower opacity. Groups of nearby points represent similar sequences which are different from the others. However, larger distances between groups should not be over-interpreted.

The result is a two-dimensional representation of the antibody sequence space, as shown in Figure 1. It can be used to visually distinguish between datasets that span a wide portion of the sequence space and datasets with a higher internal similarity.

We find that the antibodies in both the Jain and the Roche-34 dataset are generally unrelated, although there are some groups of similar antibodies in both cases. In contrast, the Roche-127 dataset has higher similarity, such that most antibodies fall into two groups, with few antibodies outside of those groups.

In Supplementary Table S4, we show the germline annotation [generated using ANARCI (Dunbar and Deane, 2016)] of the antibodies in all public datasets. Furthermore, we show a similarity matrix of all sequences in Supplementary Table S5. It shows that the Roche-127 set is significantly less diverse than the other datasets.

### 2.2 Performance of different scales

For each of our datasets, we compute the SASA score as well as the positive-SASA score of all antibodies. The scores are discussed in more detail in the Methods section. In short, the SASA score (defined as $S_{surf}=\sum_{i}^{atoms}h_{i}\times A_{i}$ ) is the sum of all solvent accessible surface areas (SASAs) $A_{i}$ of the amino acids in the Fv, multiplied by the respective hydrophobicity values $h_{i}$ . The positive-SASA score is the same, but only taking the hydrophobic amino acids into account.

**FIGURE 2:**
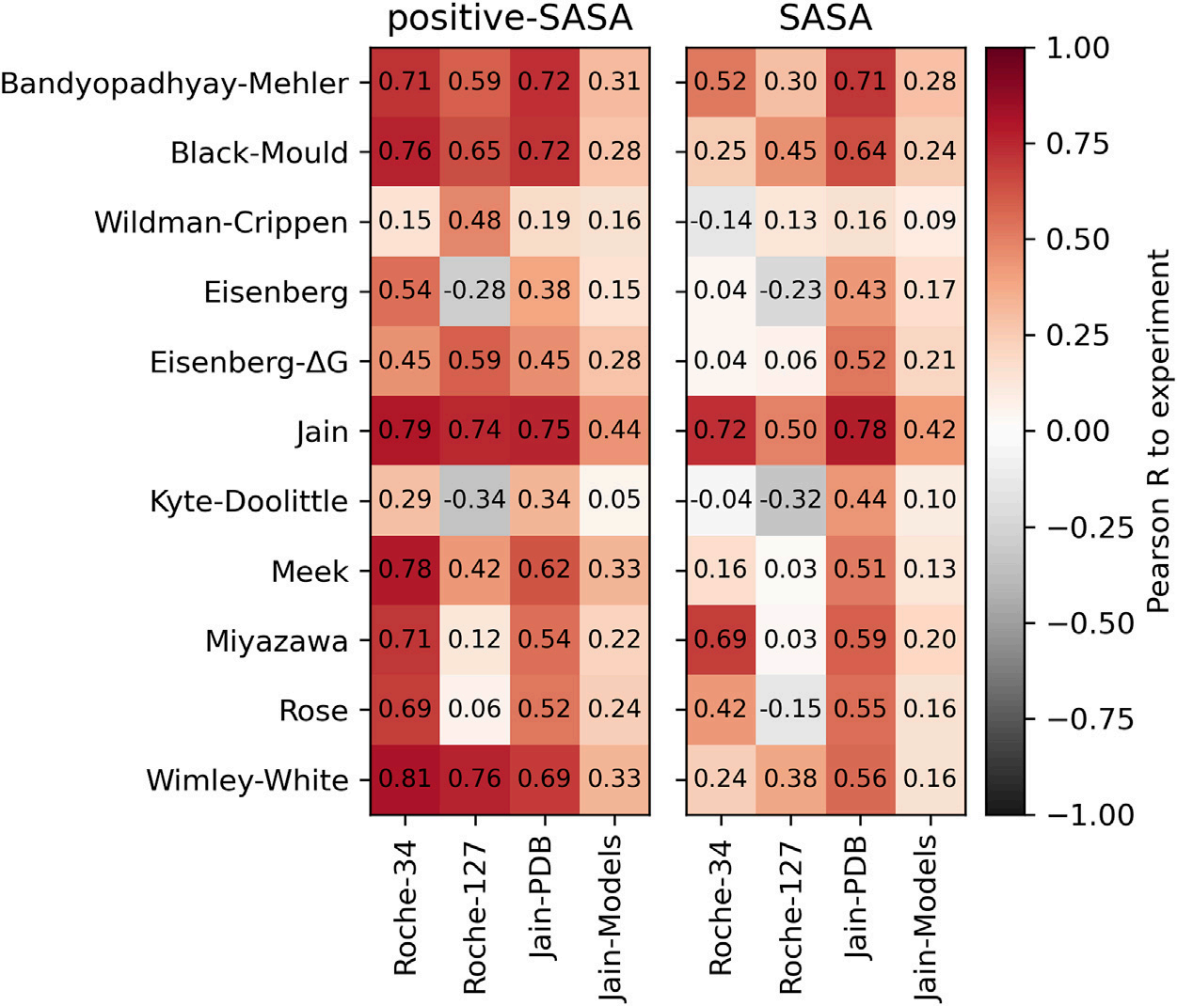
Pearson correlation values obtained by applying the positive surface score (left) and the direct surface score (right) on the different datasets and comparing to the experimental HIC data.

In the left panel of Figure 2, we show the Pearson correlation between the positive-SASA score and the respective experimental HIC values. We find good correlations using hydrophobicity scales that are optimized towards HIC or other RP-HPLC data, such as the Jain or Meek scales. Furthermore, we find good correlation values using the Wimley-White scale. The Miyazawa scale, which has often been associated with HIC prediction in literature, performs well on the Roche-127 set but not on the Roche-34 set. The relatively old scales by Eisenberg as well as Kyte and Doolittle do not perform well in our analysis. Furthermore, we find significantly worse predictivity of all scales when using homology models in the Jain-Models dataset.

In the right panel, we show the same analysis using the total surface score. While the general trends are the same, the overall correlation is significantly lower. The best-performing scale in this analysis is the Jain scale, which is little surprising, since it was optimized to predict HIC retention.

### 2.3 Other methods

**FIGURE 3:**
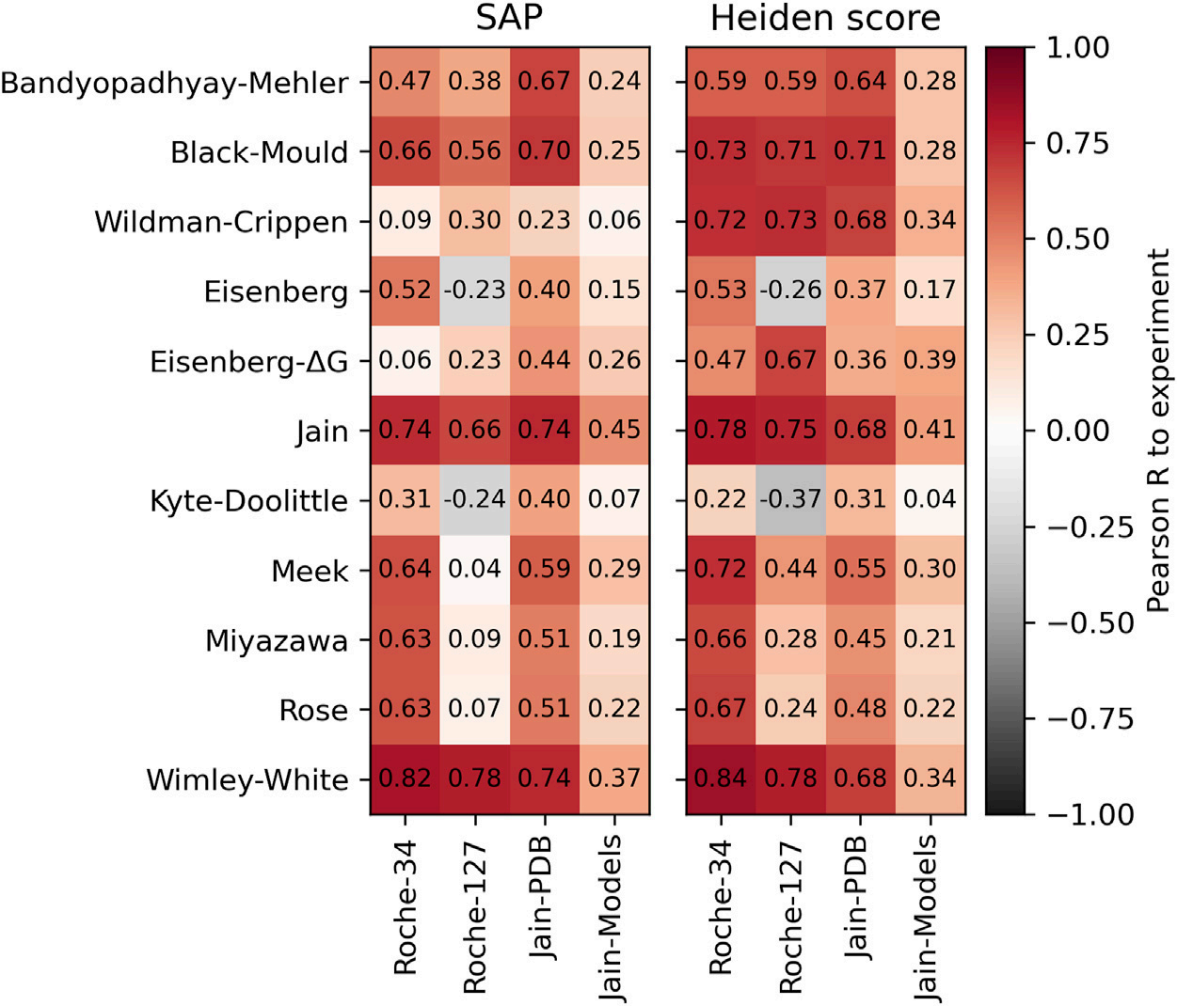
Performance of the SAP method (left) and the Heiden method (right) with different hydrophobicity scales. The Pearson correlation was calculated with respect to the HIC retention times of the respective dataset.

In addition to the simple surface scores, we created our own implementation of the Spatial Aggregation Propensity (SAP) method (Voynov et al., 2009). This method is originally used in combination with the Black-Mould hydrophobicity scale (Black and Mould, 1991), but we also combine it with several other hydrophobicity scales. In addition, we also test our own implementation of the Heiden method (Heiden et al., 1993). Since this method can reasonably work with atomic hydrophobicity scales, it is expected that the scales by Wildman and Crippen (1999) as well as the atomic hydrophobicity scale of Eisenberg and Mclachlan (1986) perform better. The result is shown in Figure 3. The scores are described in detail in the *Methods* section.

Again, we find good correlations to the experimental HIC values using the Wimley-White scale. Furthermore, both methods work reasonably well with their original scales, which is the Black-Mould scale for SAP and the Wildman and Crippen scale for the Heiden method. We note that the Heiden method works especially well with atomic hydrophobicity scales such as the Crippen or Eisenberg-ΔG scale.

While the highest predictivity is found using the Wimley-White scale, we note that atomic hydrophobicity scales have the further advantage of a higher spatial resolution, permitting more detailed analysis of the surface properties. This will be used for the spatial analysis of cavities below.

### 2.4 Performance of homology modelling packages

**FIGURE 4:**
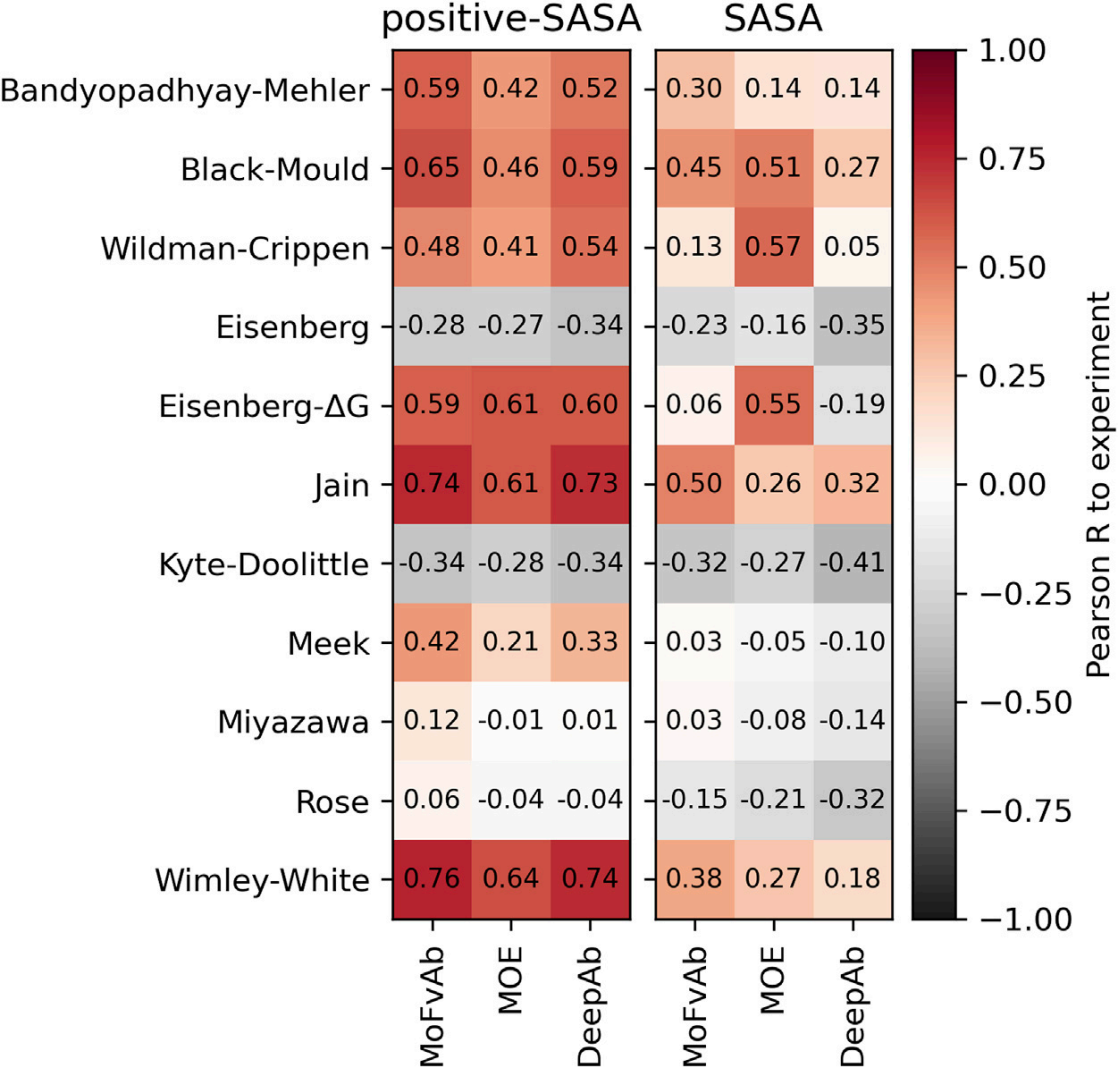
Performance of different homology modelling packages on the Roche-127 set in combination with the positive-SASA (left panel) and SASA (right panel) scores as well as different hydrophobicity scales. The Pearson correlation was calculated with respect to the HIC retention times of the Roche-127 dataset.

To compare the efficiency of different homology modelling packages, we performed our calculations on the Roche-127 dataset using models created by MoFvAb, MOE, and DeepAb, and compare the results. We find that MoFvAb and DeepAb perform very similarly, while MOE performs slightly worse in combination with most residue-based scales. However, when using the SASA score (the right panel in Figure 4), MOE performs better in combination with the atom-based Crippen and Eisenberg-ΔG scales, as well as the residue-based Black-Mould scale. This might originate from a different side chain packing of MOE compared to the other modelling packages.

### 2.5 Effect of sampling through molecular dynamics

**FIGURE 5:**
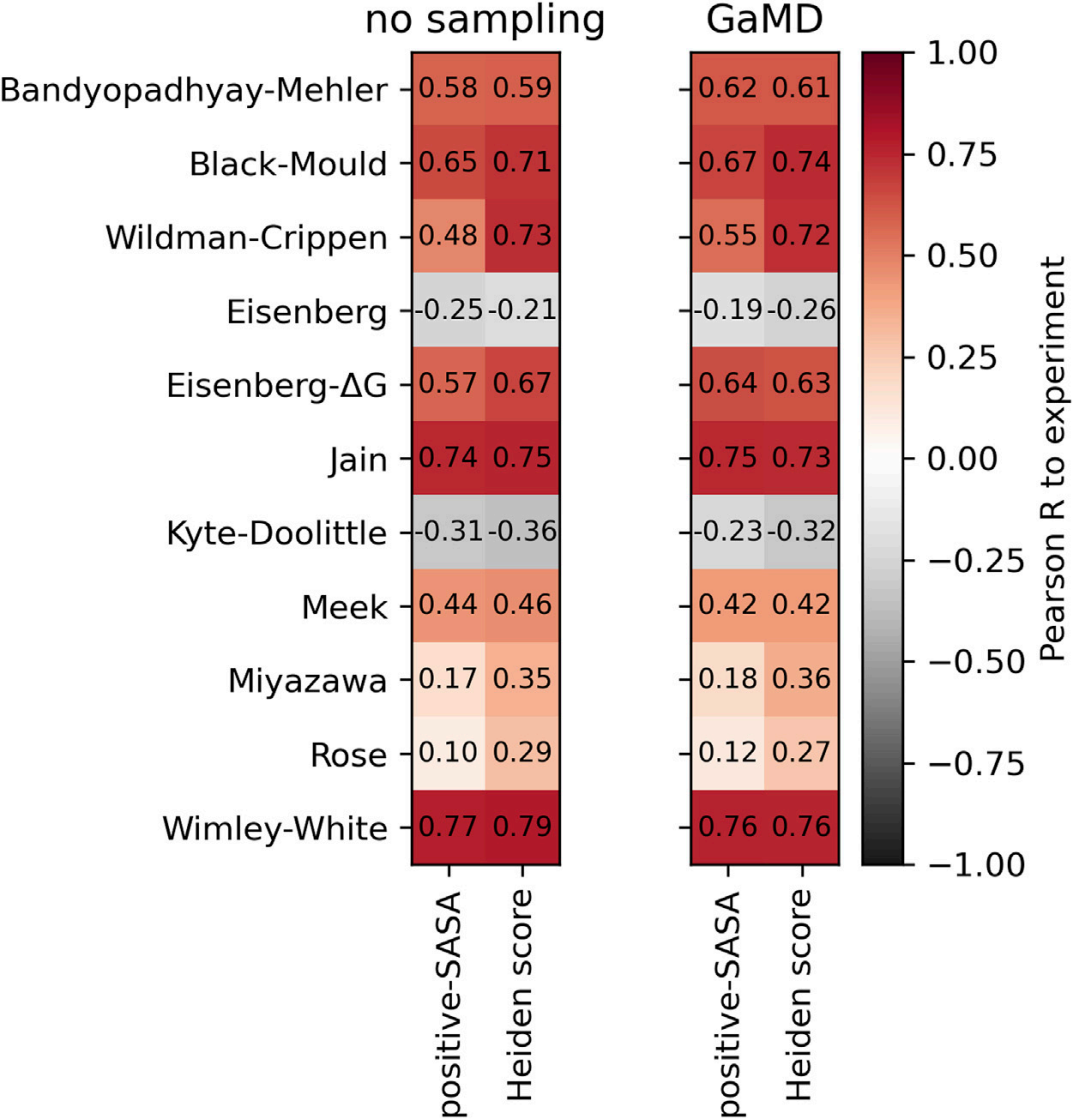
Comparison of the performance of the positive-SASA score using the MoFvAb models directly (left panel) and using an average over 200 ns of GaMD simulation (right panel). In each panel, the left column shows the positive-SASA score, while the right column shows the Heiden score. The Pearson correlation was calculated with respect to the HIC retention times of the Roche-127 dataset.

To test whether sampling of conformational ensembles through short molecular dynamics simulations improves the description of hydrophobicity, we performed 200 ns Gaussian accelerated Molecular Dynamics (GaMD) (Miao et al., 2015) simulations of each antibody in the Roche-127 set, using the MoFvAb models as starting structures. We then computed the positive-SASA score and the Heiden score and compare them to the experimental values. The result is shown in Figure 5.

As in our previous work (Waibl et al., 2021), we find no systematic improvements of the predictivity due to the sampling. The difference between hydrophobicity scales is clearly bigger than that due to the conformational sampling. We find improvements in the atomic Wildman-Crippen and Eisenberg-ΔG scales, while the effect on most residue-based scales is small. This suggests that the sampling of sidechain conformations might be better than that of global motions within the protein.

### 2.6 Cavity effects

**FIGURE 6:**
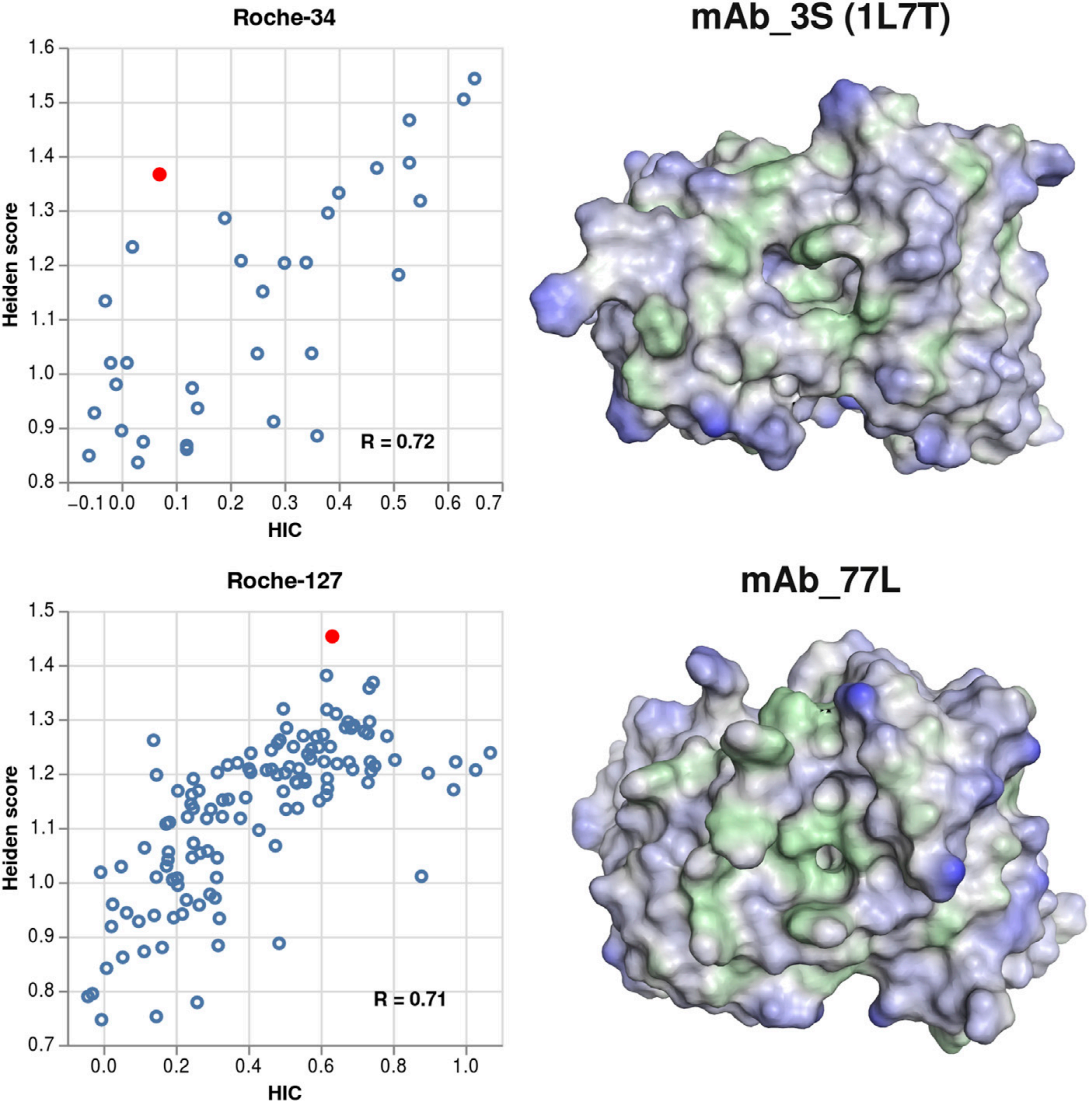
Examples for structures where cavities lead to over-prediction of the surface hydrophobicity. Left column: scatter plots showing the Heiden score vs. the experimental HIC values, for the Roche-34 set (top) and the Roche-127 set (bottom). In each plot, one antibody is marked, and the respective surface is shown at the right side. Green surface corresponds to hydrophobic regions (Heiden score > 0) and blue surface corresponds to hydrophilic regions (Heiden score < 0).

When visualizing structures of antibodies where the predicted hydrophobicity exceeds the experimental one, we often find that the structures contain cavities or pockets. Those cavities can represent, for example, binding pockets of antibodies that bind small molecules, or they can occur due to unfavorable sidechain packing in the homology modelling process. In Figure 6, we show examples of a chemically meaningful and an erroneous example of cavities, highlighting the respective structures in the correlation plots.

The 1L7T crystal structure (Valjakka et al., 2002) contains the unbound structure of an anti-testosterone Fab fragment. The binding pocket is clearly visible, albeit smaller than in the corresponding bound structure (PDB code 1VPO). While the 1L7T structure is scored very high by the Heiden method, the low experimental HIC retention time indicates that a portion of the hydrophobic surface (probably the binding pocket) is inaccessible to the HIC column.

In the MoFvAb model of mAb_3L, the CDR regions are modelled as a very rugged surface, with several small cavities and hydrophobic residues pointing towards the solvent. However, this might be due to non-optimal side chain packing in the homology modelling process. Again, comparison to the experimental values indicates that not all regions of the modelled hydrophobic surface are exposed to the HIC column.

**FIGURE 7:**
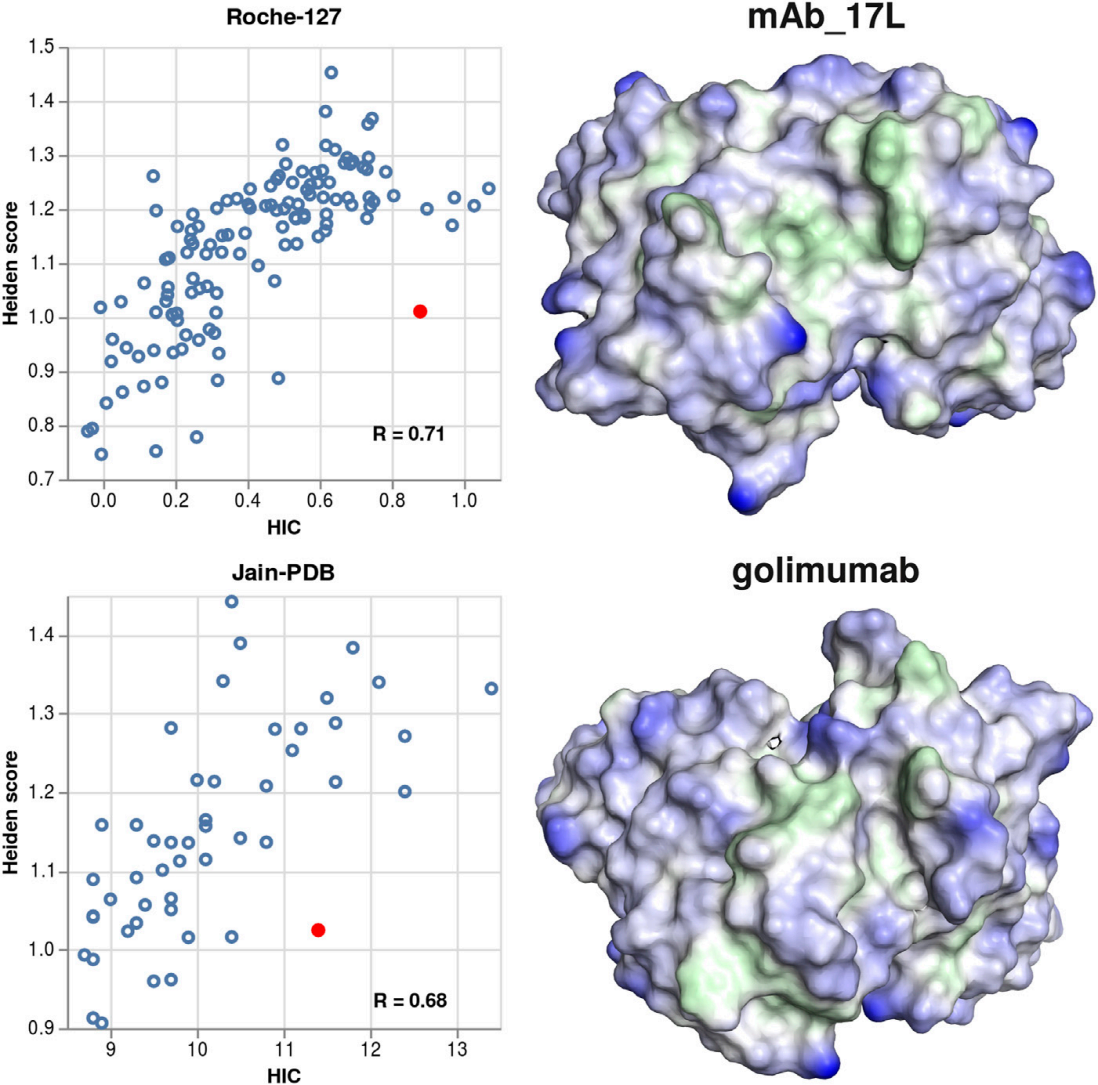
Examples for structures where the experimental hydrophobicity is under-predicted by the Heiden score. Left column: scatter plots showing the Heiden score vs. the experimental HIC values, for the Roche-127 set (top) and the Jain-PDB set (bottom). In each plot, one antibody is marked, and the respective surface is shown at the right side. Green surface corresponds to hydrophobic regions (Heiden score > 0) and blue surface corresponds to hydrophilic regions (Heiden score < 0).

We also visualized several antibodies where the predicted hydrophobicity is significantly lower than the experimental one, to check whether they would show a particularly smooth surface. In contrast to this expectation, we find several instances of broad cavities, which might be able to fit a phenyl or butane sidechain of the HIC stationary phase. Two examples (mAb_17L and golimumab) are shown in Figure 7.

## 3 Discussion

In the cases we studied, it is always better to predict HIC retention based on only the positive amino acid contributions. Even the Jain scale performs worse using the total surface score instead of the positive surface score. The only exception is the Jain dataset in combination with the Jain scale, which is not surprising since this scale is optimized to predict HIC retention. However, the Jain scale performs significantly worse on other datasets when using the SASA score (which includes the negative hydrophobicity values), which indicates poor transferability of the hydrophilic values. However, the transferability of hydrophobic values seems to be better, since the positive-SASA score using the Jain scale performs well also on the Roche-34 and Roche-127 datasets. We find a similar trend also for other hydrophobicity scales, which indicates that the poor transferability of hydrophilic values in HIC prediction is a general phenomenon, while the hydrophobic values seem more robust.

This finding is consistent with the idea that protein-column interactions in HIC are dominated by the most hydrophobic surface regions (Mahn et al., 2005). We have shown previously that the charged amino acids form very strong enthalpic interactions with the surrounding water (Schauperl et al., 2016). However, these amino acids do not have a large impact on HIC retention, since they keep their hydration shell when the protein binds to a HIC column, while only the more hydrophobic amino acids are dehydrated.

We therefore conclude that HIC measurements are controlled essentially by the most hydrophobic surface regions. This is consistent with previous findings by Mahn et al. (2005), who achieved high predictivity using a molecular docking approach to identify the most probable interaction region on the surface of ribonucleases, and scored them by the hydrophobicity of this region. We expect that this interaction region frequently coincides with a strongly hydrophobic surface region.

When visualizing antibodies where the hydrophobicity is over-predicted compared to experiment, we sometimes find cavities in the surface. These cavities can be, for example, binding pockets of antibodies that bind to small molecules, or they can be due to inaccurate sidechain packing in the homology models. In both cases, they lead to an increased hydrophobic surface. Since this is not reflected by a higher experimental HIC retention time, we assume that those pockets do not interact with the stationary phase.

We also show two examples of antibodies where the hydrophobicity is under-predicted compared to the experiment. In contrast to the over-predicted antibodies, the cavities found in those structures are broader or not very deep. We expect that such broader hydrophobic cavities can form specific interactions with the stationary phase, thus leading to a high HIC retention time although there is only a relatively small hydrophobic surface. This finding is also consistent with the idea that binding to HIC columns is dominated by the most hydrophobic region.

In general, methods based on the solvent-accessible surface area (SASA) perform rather poorly in combination with atomic hydrophobicity scales. This is expected due to the underlying assumption of additivity of the hydrophobic contribution: Correlations between the hydrophobic effect of neighboring atoms are likely stronger than those between neighboring residues. The surface projection by Heiden et al. (1993) provides a smoother way of projecting hydrophobicity scales onto the protein surface, leading to an improvement in combination with atomic scales.

Our results using the Spatial Aggregation Propensity (SAP) algorithm are similar to those using the positive-SASA score. While we used a cutoff radius R of 5 Å throughout the main text, Supplementary Figure S1 shows the comparison between R of 5 or 10 Å. In almost all cases, the cutoff of 5 Å performs better.

We find that homology models generated with MoFvAb and DeepAb perform very similar in terms of predicting experimental HIC values. On the other hand, models generated by MOE perform slightly worse in combination with most residue-based hydrophobicity scales, while outperforming them when combined with atomic scales. One possible explanation would be that MOE is more accurate at predicting side chain orientation, while large-scale contributions to the structure, such as inter-domain orientation, are predicted better by MoFvAb and DeepAb.

Our findings show that it is most difficult to work with homology models of highly diverse data sets. Several hydrophobicity scales provide good correlations with the Roche-127 dataset, which consists of homology models with high sequence similarity. We also find good correlations using the Jain-PDB set and the Roche-34 set, which have large sequence diversity but consist mostly of crystal structures. Only the HIC data of the Jain-models set is poorly predicted, likely because there is a high sequence diversity combined with homology models, which are less reliable than crystal structures. This means that there are many differences between structures, while the SASA of each amino acid is not completely reliable. In that case, summation of errors can lead to large errors in the total hydrophobicity score.

We expect the CDR-H3 loop to be the most challenging part of the structure to predict. Despite the enormous recent advances in predicting antibody structures (Ruffolo et al., 2021; Abanades et al., 2022), this loop remains difficult to predict accurately due to its high flexibility and unchallenged diversity in length, sequence, and structure. As the CDR-H3 loop is situated in the center of the antibody binding site, it influences the conformations of the neighboring CDR loops and consequently plays a critical role for predicting and quantifying surface hydrophobicity (Regep et al., 2017; Fernandez-Quintero et al., 2020).

The agreement between hydrophobicity scores and HIC retention times depends strongly on the hydrophobicity scale. The Kyte-Doolittle and Eisenberg scales perform poorly, probably because they were aimed at quantities different from HIC retention times. In contrast, the Jain scale, which is parameterized to predict HIC retention times, performs much better. Furthermore, the Wimley-White scale, which represents the free energy change when transferring pentapeptides between water and a lipid bilayer, also performs very well. A common feature of the Jain and Wimley-White scales is that they assign high hydrophobicity to aromatic residues and especially to tryptophan.

The Meek and Miyazawa scales perform comparably well on datasets which contain crystal structures (Roche-34 and Jain-PDB), but not on datasets that contain only homology models (Roche-127 and Jain-models). This contrasts with the better predictions obtained using the Jain and Wimley-White scales. The reason might be related to differences in the amino acid composition of the datasets, but also to the lower hydrophobicity assigned to tryptophan and tyrosine in the Meek and Miyazawa scales. Especially tyrosine is often found in antibody CDR regions. Since the CDRs also encompass the highest sequence diversity within an antibody, their amino acid composition is a main contributor to their surface properties (Yugandhar and Gromiha, 2014).

Thus, we conclude that several factors must be considered to predict HIC-based hydrophobicity using hydrophobicity scales. The choice of the hydrophobicity scale is crucial. There are several scales which correlate well with experimental HIC data. However, the Kyte-Doolittle and Eisenberg scales produce very poor results, even though they are still widely used. Furthermore, it is important to be aware about the sequence diversity of the dataset in question, as well as the reliability of the available structures. When predicting the hydrophobicity of highly diverse antibodies, very reliable structures—such as crystal structures—are required, while less diverse datasets can be effectively described by homology models.

## 4 Theory and methods

### 4.1 Hydrophobicity scales

Scales were normalized by adding a constant such that Gly has a value of zero and scaled such that hydrophobic residues are positive and the variance of the values is 1. The variance was calculated as the average of $h^{2}$ , where h denotes the individual values relative to Gly. The resulting values are shown in Table 2.

**TABLE 2:** The per-residue hydrophobicity scales that were used in this work.

Residue	BaMe	BlMo	Ei	KyDo	Me	Ro	WiWh	Ja	Mi
ALA	0.75	0.37	0.15	0.76	0.07	0.18	−0.20	0.06	0.40
ARG	−0.02	−1.52	−3.09	−1.41	0.11	−0.71	−0.41	−0.32	−0.15
ASN	−0.16	−0.79	−1.29	−1.06	0.11	−0.80	−0.51	0.13	−0.37
ASP	−0.50	−1.43	−1.42	−1.06	−1.08	−0.89	−1.53	−0.43	−0.43
CYS	2.60	0.55	−0.19	1.00	−0.90	1.69	0.31	0.55	1.65
GLN	−0.11	−0.76	−1.37	−1.06	−0.63	−0.89	−0.71	0.46	−0.29
GLU	−0.54	−1.40	−1.26	−1.06	−2.23	−0.89	−2.51	−0.72	−0.40
GLY	0.00	0.00	0.00	0.00	0.00	0.00	0.00	0.00	0.00
HIS	0.57	−1.00	−0.90	−0.96	−0.46	0.53	−0.20	0.03	0.30
ILE	2.19	1.34	0.92	1.68	1.83	1.42	0.35	1.54	2.08
LEU	1.97	1.34	0.60	1.44	1.16	1.16	0.71	1.54	1.91
LYS	−0.90	−0.67	−2.03	−1.20	0.01	−1.78	−0.59	−1.07	−0.74
MET	1.22	0.73	0.16	0.79	0.63	1.16	0.30	0.49	2.14
PHE	1.92	1.52	0.73	1.10	1.74	1.42	1.43	2.48	2.18
PRO	0.72	0.64	−0.37	−0.41	0.80	−0.71	−0.55	0.44	−0.29
SER	0.11	−0.43	−0.68	−0.14	0.16	−0.53	−0.15	0.07	−0.19
THR	0.47	−0.15	−0.55	−0.10	0.36	−0.18	−0.16	0.16	0.00
TRP	1.51	1.16	0.34	−0.17	1.96	1.16	2.33	2.81	1.52
TYR	1.36	1.16	−0.23	−0.31	0.80	0.36	1.19	1.84	0.68
VAL	1.88	1.00	0.61	1.58	0.36	1.25	−0.08	0.97	1.51

Abbreviations: BaMe, Bandyopadhyay-Mehler; BlMo, Black-Mould; Ei, Eisenberg; KyDo, Kyte-Doolittle; Me, Meek; Ro, Rose; WiWh, Wimley-White; Ja, Jain; Mi, Miyazawa.

### 4.2 Datasets

The following datasets were investigated in this work:

#### 4.2.1 Roche-34

This dataset contains 34 antibodies for which HIC measurements of full-length IgGs have been performed. Of this dataset, 14 have crystal structures deposited in the PDB (Berman et al., 2000). Further three antibodies have been previously published and have a name in the standard antibody nomenclature, but do not have crystal structures. The other 17 structures are Roche-internal. The relative HIC retention times of this dataset, as well as identifiers and sequences of the published antibodies, are shown in the Supplementary Table S1.

#### 4.2.2 Roche-127

This dataset contains 127 Roche-internal antibodies for which HIC measurements of full-length IgGs have been performed. Since no crystal structures are available for those antibodies, homology models were created using MoFvAb, as described below. Relative HIC retention times are shown in Supplementary Table S2.

#### 4.2.3 Jain-PDBs

This dataset is a subset of the publicly available dataset by Jain et al. (2017b), containing 49 structures for which crystal structures are available from the PDB. The HIC measurements from the original publication were used as reference data for this dataset. The used PDB codes are shown in Supplementary Table S3.

#### 4.2.4 Jain-Models

This dataset contains 77 antibodies from the dataset by Jain et al. (2017b), for which no crystal structures were found in the PDB. Homology models were created using DeepAb (Ruffolo et al., 2021).

### 4.3 tSNE

tSNE (t-distributed stochastic neighbor embedding) (van der Maaten and Hinton, 2008) is a dimensionality reduction technique that aims to preserve the local structure, i.e., the distance information between close-lying data points. Here, we apply it to generate a two-dimensional representation of the antibody sequence space, based on a distance matrix generated using Clustal Omega (Sievers et al., 2011; Sievers and Higgins, 2018; Sievers et al., 2020). We used the tSNE implementation in Scikit-Learn (Pedregosa et al., 2011) with a “perplexity” setting of 500. The value of 500 was chosen because it is significantly bigger than the size of the Roche-127 set but small compared to the total number of datapoints. If there are groups of similar antibodies with a size larger than the perplexity, the relation of this group to other groups is lost almost completely.

While t-SNE excels at reproducing the relation between adjacent datapoints (i.e., similar sequences) in the two-dimensional embedding, the relations between more distant sequences are less reliable. It has been shown that the initialization method is crucial to obtain embeddings that also reproduce some of the global structure (Kobak and Linderman, 2021). While literature states (Kobak and Linderman, 2021) that initialization using Laplacian Eigenmaps (LE) (Belkin and Niyogi, 2002) is superior to random initialization at preserving global structure, this approach performed very poorly for our dataset, producing consistently lower Pearson correlations between the original distance matrix and the two-dimensional distances. We therefore chose to use random initialization, but repeat the calculation 10 times and use the best embedding as judged by the Kullback-Leibler divergence (Kullback and Leibler, 1951).

### 4.4 Starting structures

For the Jain-PDB dataset, equilibrated versions of the crystal structures of the Jain dataset were taken from our previous work (Waibl et al., 2021).

All homology models were created starting from the respective VH and VL sequences. The program settings were as follows:

#### 4.4.1 MoFvAb

The settings for MoFvAb were as in the original work by Bujotzek et al. (2015).

#### 4.4.2 MOE

Fab Models were created using the antibody modeling protocol in MOE 2020.09. Templates were selected automatically from the built-in database, and the resulting structures were extended to FAb fragments. MOE models were used as exported from MOE. All protonation states were kept the same, histidines with a hydrogen in δ-position were renamed to HID, and histidines with a hydrogen in the ε-position were renamed to HIE. CYS residues in a disulfide bond were renamed to CYX. Hydrogen atoms were removed and re-added in standard positions using the reduce program (Word et al., 1999).

#### 4.4.3 DeepAb

DeepAb (Ruffolo et al., 2021) was downloaded from GitHub at 3 Nov 2021, and used with PyRosetta 4 release 293 (Chaudhury et al., 2010). The pre-trained model was used, and calculations were run without GPU acceleration. All settings were left at their default values. The protonation was kept as in the DeepAb output, histidines and cysteines were renamed in the same way as for the MOE models. We note that the protonation only affects the atomic hydrophobicity scales, since no simulations were performed starting from the DeepAb models.

### 4.5 Gaussian accelerated molecular dynamics simulations

GaMD simulations were performed starting from the MoFvAb models of the Roche-127 dataset. C_H_1/C_L_ domains were added for the simulations but omitted in all analyses.

The models were protonated using the Protonate3D protocol (Labute, 2009) in MOE (Chemical Computing Group ULC, 2020) to obtain consistent protonation patterns.

Gaussian accelerated Molecular Dynamics (GaMD) (Miao et al., 2015) simulations were performed using the same protocol as described previously (Waibl et al., 2021). The ff14SB force field (Maier et al., 2015) was used in combination with the TIP3P water model (Jorgensen et al., 1983). Energy statistics for GaMD were collected during a number of MD frames equal to 4 times the number of atoms in the system, rounded up to the next picosecond (Case et al., 2019). Then, GaMD was run using a dual boost. SHAKE (Ryckaert et al., 1977) was used on all bonds including hydrogen. The integration timestep was 2 fs, using a Langevin thermostat (Adelman and Doll, 1976) at 300 K with a collision frequency of 2 ps^−1^ and a Monte Carlo barostat (Åqvist et al., 2004) with one volume change attempt per 100 steps.

For post-processing, one frame per nanosecond was used, resulting in 200 representative frames. Energies were collected every picosecond and were used to reweight the probabilities of representative frames using cumulative expansion to the second order (Miao et al., 2014).

### 4.6 Hydrophobicity scores

All scores were calculated in Python using a series of in-house Python scripts.

Residue-based hydrophobicity scales were assigned based on the residue name. All protonation states of histidine were considered equally except for scales that contain separate values.

The atoms in each of the 20 amino acids were assigned types according to the Crippen scale using the SMILES matching functionality in RDKit (Landrum et al., 2020). The hydrogen atoms bound to aromatic nitrogen in TRP and HIS residues were set to the H3 type (“amine”), rather than H2 (“alcohol”). All other types were used as assigned by RDKit. After the initial assignment, atom types were assigned by a simple lookup table using the residue name and atom name as a key.

The solvent-accessible surface area (SASA) was computed using the Shrake-Rupley algorithm (Shrake and Rupley, 1973) implemented in MDTraj (McGibbon et al., 2015).

The direct surface score *S*_*surf*_ was computed by multiplying the SASA of each atom *A*_i_ by the hydrophobicity value obtained from a scale, *h*_*i*_. This score is conceptually consistent with an experiment where all surface-exposed residues are desolvated, thereby contributing to the overall hydrophobicity.

$$\begin{array}{l} S_{surf}=\overset{atoms}{\underset{i}{\sum }}h_{i}\times A_{i} \end{array}$$ (1)

The positive surface score *S*_*pos*_ was defined in the same way, except that all negative *h*_*i*_ values were set to zero. This is consistent with an experiment where only the hydrophobic surface regions are desolvated, while the hydrophilic regions remain in contact with water. It is expected (Mahn et al., 2009) that this matches the experimental conditions of HIC more closely.

The Spatial Aggregation Propensity (SAP) (Chennamsetty et al., 2010) was computed as:

$$\begin{array}{l} SAP_{i}=\overset{side\;chain\;atoms}{\underset{j,r_{ij}<R}{\sum }}\frac{A_{j}}{A_{j}^{residue}}h_{j} \end{array}$$ (2)

where *r*_*ij*_ is the distance between atoms *i* and *j*, and *R* is the cutoff radius, chosen as 5 Å in this study to be consistent with the original work. $A_{j}^{residue}$ is the average sidechain SASA of a residue capped with N-methyl and acetyl groups in TIP3P water, computed from a 100 ns cMD simulation using the ff14SB Amber force field and TIP3P. The SASA was again computed using MDTraj.

The resulting hydrophobicity score was computed as:

$$\begin{array}{l} S_{SAP}=\overset{atoms}{\underset{i}{\sum }}\max\left(SAP_{i},\;0\right) \end{array}$$ (3)

The final *SAP* score is computed including atoms that are not solvent-exposed. However, the solvent-exposure is already considered by using $A_{j}$ in the individual atom scores. This is consistent with the original literature (Chennamsetty et al., 2009; Lauer et al., 2012).

For the score by Heiden et al. (1993), we first computed a solvent-excluded surface (SES), using the post-processing routines of our previous work (Waibl et al., 2022) (available from https://github.com/liedllab/gisttools) to compute the lowest possible distance to a solvent molecule on a 3D grid, and then creating an isosurface at the solvent radius of 1.4 Å using the marching cubes algorithm in scikit-image (van der Walt et al., 2014). For each vertex k of the surface, we compute the molecular lipophilicity potential (MLP) using:

$$\begin{array}{l} MLP_{k}=\frac{\overset{atoms}{\underset{j,r_{jk}<R}{\sum }}g\left(r_{jk}\right)h_{j}}{\overset{atoms}{\underset{j,r_{jk}<R}{\sum }}g\left(r_{jk}\right)},\;g\left(r\right)={\left[\exp\left(\alpha \left(r-\frac{R}{2}\right)\right)+1\right]}^{-1} \end{array}$$ (4)

This is essentially a weighted average over nearby atoms, with *g*(*r*) as the weighting function. *g*(*r*) is a (mirrored) logistic function with height 1, steepness *α* and midpoint *R*/2, where *R* is the cutoff radius. Consistent with the original implementation, we choose *α* as 1.5 Å^−1^ and *R* as 5 Å. We compute a hydrophobicity score as

$$\begin{array}{l} S_{Heiden}=\overset{vertices}{\underset{k}{\sum }}\max\left(MLP_{k},\;0\right)\times A_{k} \end{array}$$ (5)

where *A*_*k*_ is the SASA of vertex *k*, which is calculated by splitting the area of each triangle to the three vertices. The sum over all positive vertex scores is used to define the Heiden score. By visualizing the hydrophobic surface regions, we find the same hydrophobic patches as defined by MOE. The patch area, however, is not the same due to slight differences in the definition of the molecular surface and the way the patches are searched.

### 4.7 Hydrophobic interaction chromatography

Apparent hydrophobicity was determined essentially as described previously (Jarasch et al., 2015), by injecting 20 µg of sample onto a HIC-Ether-5PW (Tosoh) column equilibrated with 25 mM Na-phosphate, 1.5 M ammonium sulfate, pH 7.0. Elution was performed with a linear gradient from 0 to 100% buffer B (25 mM Na-phosphate, pH 7.0) within 60 min. Retention times were compared to protein standards with known hydrophobicity.

## Data Availability

The original contributions presented in the study are included in the article/Supplementary Material, further inquiries can be directed to the corresponding author.

## Supplementary Material

Supplementary Table S1 Relative HIC retention times for the antibodies of the Roche-34 dataset. For publicly available antibodies, the sequences and PDB codes are also reported.

SUPPLEMENTARY TABLE S2 Relative HIC retention times for the antibodies of the Roche-127 dataset.

SUPPLEMENTARY TABLE S3 PDB codes that were used for the Jain dataset. Where no PDB code is shown, homology models are shown. HIC data and sequences were obtained from (Jain et al. 2017b).

SUPPLEMENTARY TABLE S4 Germline annotation for all investigated antibodies.

SUPPLEMENTARY TABLE S5 Similarity matrix of all investigated antibodies.
