# Supplementary material for: Comparison of hydrophobicity scales for predicting biophysical properties of antibodies
Source: Front Mol Biosci. 2022 Aug 31;9:960194. doi: 10.3389/fmolb.2022.960194 (PMC9475378; doi:10.3389/fmolb.2022.960194)
Supplement: Supplementary file 1 [file DataSheet2.docx]

Supporting Information

For the Manuscript

Comparison of Hydrophobicity Scales for Predicting Biophysical Properties of Antibodies

Franz Waibl^1^, Monica L. Fernández-Quintero^1^, Florian S. Wedl^1^, Hubert Kettenberger^2^, Guy Georges^2^, Klaus R. Liedl^1*^

1: Department of General, Inorganic and Theoretical Chemistry, University of Innsbruck, Innsbruck, Austria

2: Roche Pharma Research and Early Development, Large Molecule Research, Roche Innovation Center Munich, Penzberg, Germany

* to whom correspondence should be addressed: Klaus.Liedl@uibk.ac.at

# Distance Cutoff for the Spatial Aggregation Propensity

In the original publications regarding the Spatial Aggregation Propensity (SAP) (Chennamsetty et al., 2009; Lauer et al., 2012), different values for the cutoff radius *R* of 5 or 10 Å were explored. In the main text of this manuscript, we only used a 5 Å cutoff. In SI Figure 1, we compare our results between the two cutoff values, for all datasets investigated in this work.


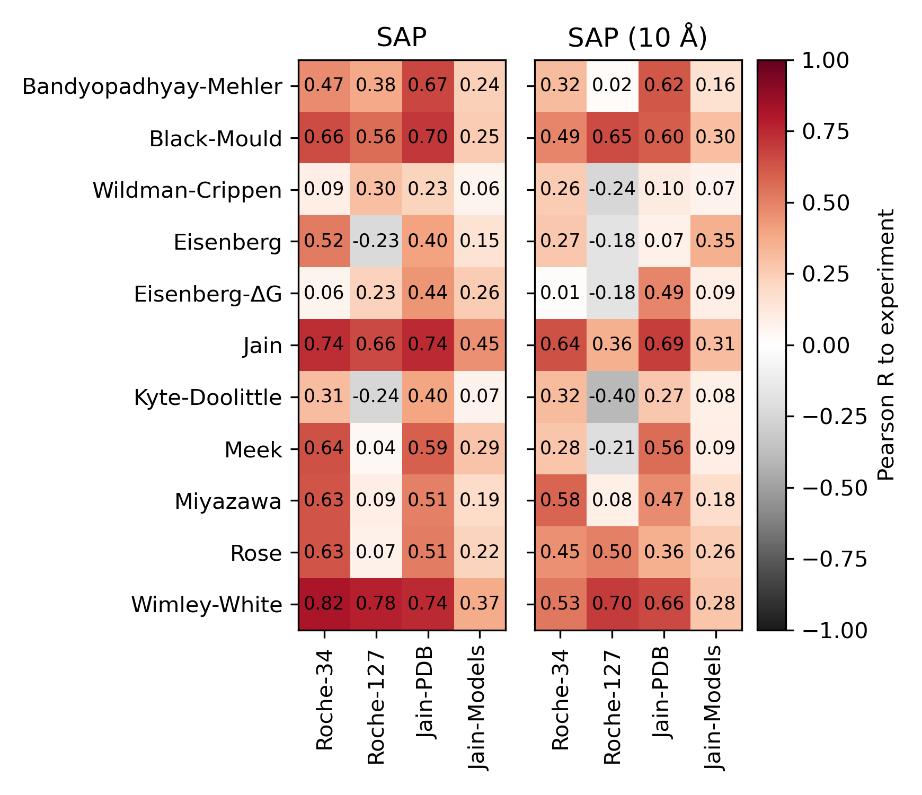


SI Figure 1: Performance of the SAP method using 5 Å (left) and 10 Å (right) as a cutoff, with different hydrophobicity scales. The Pearson correlation was calculated with respect to the HIC retention times of the respective dataset.

We consistently find worse correlations to the experimental values using a 10 Å cutoff. The only exception is the combination of the Black and Mould scale (which was originally used with the SAP method) and the Roche-127 dataset, which has the highest sequence similarity. In this case, we find a minor improvement of the correlation from 0.56 to 0.65 due to the 10 Å cutoff radius. However, we believe that a cutoff of 5 Å is generally better suited for the SAP method.

# References

Chennamsetty, N., Voynov, V., Kayser, V., Helk, B., and Trout, B.L. (2009). Design of therapeutic proteins with enhanced stability. *Proceedings of the National Academy of Sciences of the United States of America* 106(29)**,** 11937-11942. doi: 10.1073/pnas.0904191106.

Lauer, T.M., Agrawal, N.J., Chennamsetty, N., Egodage, K., Helk, B., and Trout, B.L. (2012). Developability index: A rapid in silico tool for the screening of antibody aggregation propensity. *Journal of Pharmaceutical Sciences* 101(1)**,** 102-115. doi: 10.1002/jps.22758.
